# A Carnegie perspective on intermittent risk taking in entrepreneurship

**DOI:** 10.3389/fpsyg.2023.1167243

**Published:** 2023-12-27

**Authors:** Ji-hyun Kim, Ann Terlaak, Naryoung Yu

**Affiliations:** ^1^Yonsei School of Business, Yonsei University, Seoul, Republic of Korea; ^2^Wisconsin School of Business, University of Wisconsin, Madison, WI, United States; ^3^Ivey Business School, Western University, London, ON, Canada

**Keywords:** risk taking, entrepreneurial decision-making, serial entrepreneurship, experiential learning, vicarious learning, computational modeling

## Abstract

Varying risk-taking tendency is an important area of inquiry for the Carnegie perspective. Drawing on organizational learning literature, we develop a model to illuminate the mechanisms that can underlie time-varying risk taking tendency in entrepreneurship. In particular, we delineate conditions under which abrupt risk taking punctuates periods of risk-avoiding behaviors, a pattern that we call “intermittent risk taking.” We use serial entrepreneurs whose bouts with risk taking are often depicted as driven by an entrepreneurial itch to illustrate our model. In our conceptualization, decision makers engage in an interplay of experiential and vicarious learning as they move into and out of higher-risk self-employment (i.e., venture creation) with in-between stints in lower-risk wage-employment. Using a computational model to simulate the dynamics of this conceptualization, we find that vicarious learning from satisfied risk-avoiding peers can exert a pull that draws disappointed entrepreneurs into periods of risk avoidance (i.e., wage-employment). However, the moment that the satisfaction of these peers fails to convince, this pull wanes. In effect, the entrepreneur vicariously learns that the grass may not be greener on the other side which then leads them to return into self-employment. The itch for risk taking then recurs not necessarily because risky venture creation offers higher payoffs than risk-avoiding options but because decision makers come to see that risk avoidance may not be a satisfactory alternative either — a conceptualization that adds nuance to prior notions of varying risk tendencies and serial entrepreneurship.

## 1 Introduction

Risk taking is a central area of inquiry for several schools of economic and managerial thought, including the Carnegie perspective (Cyert and March, 1963). In part, it owes this centrality to its role in shaping economic growth and fueling innovation. From a macroeconomic perspective, economic entities’ risk perceptions and attitudes matter for the effects of monetary policy on the overall economy (Bauer et al., 2023). From an innovation point of view, risk taking is essential for new venture creation. This is because for aspiring entrepreneurs, new venture creation is a risky endeavor, especially when compared to engaging in alternate wage-employment. Venture creation, while often generating the same *average* payoffs than wage-employment, tends to be riskier because it is associated with payoffs that exhibit much greater variations (Hamilton, 2000; Carroll et al., 2001; Miller, 2007; Åstebro et al., 2011). All too often, it can produce not only astounding successes that push technological frontiers and disrupt established processes to generate better ones but also devastating failures.

In line with the importance of risk taking for innovation and growth, scholars working in the Carnegie perspective have long sought to illuminate the mechanisms that drive decision makers to engage in or, conversely, avoid risky endeavors and practices [e.g., the “hot stove model” proposed by Denrell and March (2001) and conceptualizations of variable risk preferences by March and Shapira (1992)]. We here continue in this line of inquiry, paying special attention to one particular pattern of risk taking: that of intermittent risk taking, i.e., a pattern whereby decision makers vacillate between risk taking and avoidance. Serial entrepreneurs—entrepreneurs that repeatedly and sequentially engage in new venture-creations (Westhead and Wright, 2017)—are a case in point. To be sure, some serial entrepreneurs engage in continuous risk seeking as they go from one venture creation straight to the next.^1^ But others engage in intermittent risk taking whereby they enter into lower-risk wage-employment in between bouts with higher-risk self-employment (Hamilton, 2000; Amaral et al., 2011; Åstebro et al., 2011). Mariam Naficy, founder and CEO of *Minted*, exemplifies this. Before founding *Minted*, she founded *Eve.com*, an online makeup company. Subsequent to selling *Eve.com* for $110 million, Ms. Naficy worked as a manager at *The Body Shop* for the next 8 years, then returning into entrepreneurship to found *Minted*, an online stationery store that as of 2021 had 2,000+ employees and 1.22 billion USD in revenue (Dun and Bradstreet, 2022). As recent studies highlight, Ms. Naficy’s shifting employment pattern is not a unique phenomenon. Analyzing the career paths of 205 entrepreneurs, Koch et al. (2021, p. 8) find that 33% of the sample exhibited mixed self-employment career patterns, with entrepreneurs frequently shifting between self-employment and wage employment along with periods of unemployment and training. Feng et al. (2022, p. 205) similarly observe that “the back-and-forth movement of an entrepreneurial career across paid jobs and new ventures is indeed common.” *What, then, are the processes that underlie such intermittent risk taking?*

Illuminating the processes that result in entrepreneurs vacillating between high- and low-risk behaviors is of both empirical and theoretical importance. Empirically, 48% of entrepreneurial activity in the U.S. is attributable to serial entrepreneurs (Kelly et al., 2020). In Europe, this share is 18–30% (Plehn-Dujowich, 2010). Intermittent risk taking also exhibits itself beyond the entrepreneurship realm, with scholars typically pointing to specific events as triggers for changes in risk taking. For example, Guiso et al. (2018) found that individual investors varied their risk taking subsequent to experiencing the 2008 financial crisis. As another example, Shum and Xin (2022) found that individual drivers’ risk taking increased following near-miss accidents, with this effect lasting for a few weeks before reverting back to its original level. Together, these studies suggest that individuals do engage in intermittent risk taking, often as triggered by the conditions they experience.

From a theoretical perspective, two lines of inquiry within the Carnegie perspective address varying risk taking behaviors. The first of these lines focuses on variable risk preferences as the result of changing fortunes and shifting attention. Specifically, March and Shapira (1992, p. 172) suggest that “the level of individual or organizational risk taking is responsive to a risk taker’s changing fortune.” Similarly, in the second edition of the Behavioral Theory of the Firm, Cyert and March (1992, p. 227) note that “preferences for high variance alternatives are not constant but are responsive to changing fortune.” But even when accumulated resources are the same, risk taking may still vary as decision makers shift their attention between aspiration levels and survival points. Using a random variable to govern how attention may shift between these points, March and Shapira (1992) show that a certain combination of attention shifts can result in varying risk taking patterns over time. From this perspective, the intermittent risk taking inherent in serial entrepreneurship could then come about because of resource levels or shifting attention between survival and aspirations points.

Theories of learning and adaptive sampling offer a second, alternative view on varying risk taking (Denrell and March, 2001). This view does not make assumptions about risk preferences. Instead, risk seeking or avoidance is the result of (possibly risk-neutral) decision makers learning from, and adapting to, their own experiences and those of others (Denrell and March, 2001; Denrell, 2003). As for the risk taking consequences of decision makers learning from their own experience, Denrell and March (2001) have coined the term “hot stove effect” to describe the tendency of experiential learning to lead decision makers to become risk averse. The term references Mark Twain’s cat: Twain’s cat sat on a hot stove lid once, never to sit on it again, not even a cold one. The idea is that as decision makers choose their actions based on prior experiences, they will avoid alternatives that had poor payoffs in the past — such as a hot lid in the case of Mark Twain’s cat, or a devastating financial loss in the case of new venture creation (Cyert and March, 1963). Because a high-risk alternative, compared to a low-risk one of equal average value, more frequently has very poor payoffs, and because one very poor experience leads the decision maker to abandon that alternative, the decision maker cannot collect any further experiences that would reveal the alternative’s true value. As a result, they become risk averse, selecting into lower-risk alternatives such as wage-employment over higher-risk ones such as venture creation (March, 1996; Denrell and March, 2001; Fazio et al., 2004; Denrell, 2008).^1^ As for the risk taking consequences of decision makers learning vicariously, effects are opposite, with vicarious learning generating upwardly biased risk taking. This is driven by sampling: because decision makers tend to sample the experiences of successful others, and because the successes or payoffs of risky alternatives tend to be larger than those of equal value but lower-risk alternatives, vicarious learning inherently involves an undersampling of failure. This leads decision makers to engage in more risk taking than they would otherwise (e.g., Greve, 1995; Baum et al., 2000; Denrell, 2003).

Our theoretical approach to intermittent risk taking is aligned with this second line of inquiry—the idea that both risk seeking and risk avoidance can result from adaptive learning. By virtue of being able to account for both low- and high-risk behaviors, adaptive learning carries clear potential for explaining intermittent risk taking. What is more, this approach does not necessitate assumptions about risk preferences and their stability. This is especially attractive for explaining phenomena in the entrepreneurship realm where the debate regarding entrepreneurs’ risk preferences and whether these preferences systematically and stably differ from those of wage-employees is of yet unresolved (Brockhaus, 1980; Stewart and Roth, 2001; Miner and Raju, 2004). Yet despite this potential, we so far lack an understanding of what, exactly, the learning processes that can result in decision makers, particularly entrepreneurs, vacillating between risk seeking and avoidance may look like. In part, this gap in understanding comes about because most studies in this space analyze the risk taking consequences of decision makers engaging in each learning mode in isolation. With each learning mode engendering either risk taking *or* risk avoidance, these studies convincingly explain how a decision maker may engage in one type of risk taking over the other, but they fall short in accounting for potential switches between the two. For instance, Denrell and March’s (2001) hot stove model solely focuses on experiential learning and the risk avoidance that ensues whereas Denrell (2003) illuminates how sole vicarious learning can result in risk seeking. To be sure, some studies do begin to explore how the two learning modes and resulting risk-taking tendencies may combine, suggesting that vicarious learning and interdependent sampling (where one decision maker’s choice of action depends on both their own attitude and that of others) can attenuate the bias against risk that emerges from experiential learning (Yechiam and Busemeyer, 2006; Denrell and Le Mens, 2007). But these studies still assume that information from one mode (vicarious learning) passively adds information to the other (experiential learning) and that it is sampling, not learning, that is interdependent. This leaves unexplored how the two learning modes may interplay and what the effects of such interplay on intermittent risk taking may be—the question that we address here.

We use serial entrepreneurship to illustrate our study on the interplay between experiential and vicarious learning and resultant risk taking. As such, we address the underlying processes of the repeated transition from one employment state, self-employment, into the other state, wage-employment, and back again. Practitioners describe these transitions as triggered by an itch that comes and goes. Scott Baxter, founder and chief executive of SA Baxter, remarks that “I’m 2 years into my next project, Doolli, a next-generation internet technology company, although it is not operating yet. SA Baxter is 7 years old. I’ve got the itch again” (Baxter, 2013). Another entrepreneur, Ben Erez, recounts that “when I shut down my first startup last year, some close friends and mentors told me “Do not worry, the itch will come back” (Erez, 2015). We here develop a theoretical underpinning for when and why that itch strikes again.

Our study’s contribution to the Carnegie perspective is two-fold. First, we illuminate how intermittent risk taking can be the result of learning and adaptive sampling rather than variable risk preferences (Cyert and March, 1963; March and Shapira, 1992). In fact, our conceptualization accommodates for decision makers to be risk neutral, thereby allowing us to sidestep assumptions whether the risk preferences of decision makers that engage in higher-risk activities like entrepreneurship are systematically different from those that engage in lower-risk activities like wage-employment (Hall and Woodward, 2010; Brown et al., 2011; see section 5.4 of Parker, 2018 for a detailed review). Second, we investigate risk taking as the result of decision makers engaging in an interplay between experiential and vicarious learning. This moves the field beyond prior conceptualizations of risk taking as stemming from just one of these modes, with risk aversion having been understood as an outcome of experiential learning and risk seeking as an outcome of vicarious learning (Denrell and March, 2001; Denrell, 2003).

Our paper also makes a third contribution, this one to the field of serial entrepreneurship. In that field, research has paid particular attention to the origins of serial entrepreneurs. It suggests, for instance, that prior self-employment allows entrepreneurs to improve their capabilities, leading them to repeatedly try their hand at venture creation (Ucbasaran et al., 2009; Westhead and Wright, 2017) and that biases like comparative optimism and overconfidence drive entrepreneurs to become serial entrepreneurs (Hayward et al., 2010; Spivack et al., 2014). Yet an integral component of serial entrepreneurship, the actual transitions between self- and wage-employment, has received comparably less attention. We contribute by developing a model that explicitly addresses these transitions. This allows us to move the focus away from analyzing self-employment as status—an emphasis that also aligns with recent developments to view entrepreneurship as a transient state rather than an absorbing one (Burton et al., 2016).

We set up the remainder of the paper as follows: We next provide some conceptual background on our learning model and its application to entrepreneurship. We then develop a formal model. Subsequently, we employ a computational simulation that allows us to examine the learning dynamics and risk-taking patterns that result from experiential and vicarious learning interplaying. After that, we discuss results and offer concluding thoughts.

## 2 Conceptual background

### 2.1 Performance feedback and the interplay of experiential and vicarious learning

Performance feedback theory—a cornerstone in theories of behavioral decision making in the Carnegie perspective—suggests that as decision makers chart their course of action, they are influenced by how the performance outcomes of their prior choices compare to their aspiration levels, i.e., the reference points that separate satisfactory outcomes from unsatisfactory ones (e.g., Cyert and March, 1963; Bromiley, 1991; March and Shapira, 1992; Greve, 1995; Miller and Chen, 2004). Outcomes near aspiration levels stimulate exploitative behaviors, i.e., local search within known alternatives, whereas outcomes that fall below aspiration levels foster nonlocal exploration (e.g., Greve, 2003).

Organizational learning scholars in the Carnegie perspective have applied these insights to shed light on how experiential and vicarious learning may interplay. Baum and Dahlin (2007), for instance, employ a performance feedback logic when explaining patterns of learning in the context of railroads’ learning from train accidents. Interpreting vicarious learning as nonlocal explorative search and experiential learning as local exploitative search, they theorize and find that decision makers emphasize experiential learning when performing near aspirations levels and that these decision makers switch to vicarious learning subsequent to unsatisfactory performance outcomes. Schwab (2007) finds similar patterns when examining how experiential and vicarious learning shape baseball teams’ incremental adjustments to previously adopted farm-team systems. He shows that satisfactory performance outcomes lead teams to rely on experiential learning for adjusting farm-team sizes while unsatisfactory outcomes lead to adjustments based upon vicariously learned sizes of others’ farm-team systems. Schwab argues that this learning-mode interplay comes about as “negative performance feedback may lead an organization to question both its ability to master the innovative practice and its ability to learn from its own performance. Such uncertainty may lead organizations to rely more on simple vicarious information” (Schwab, 2007, p. 247). Clough and Piezunka (2020) find corresponding learning patterns in the context of Formula One car builders making decisions regarding buyer–supplier relationships. Finally, Aranda et al. (2017) document a similar mechanism in the context of organizational goal setting. These authors find that when setting targets, unfavorable performance weakens the organizational unit’s reliance on its past performance; in effect, the unit relies less on experiential learning. Similar to the other studies, Aranda et al. (2017, p. 1194) argue that this comes about because “failures question existing assumptions about cause–effect relationships, which forces organizations into non-local searches… Learning from failure leads to a focus on outside organizations’ performance.”

As the above studies suggest, underlying the notion of decision makers engaging in vicarious learning in response to disappointing experiences is the idea that unexpectedly poor outcomes can lead to reflection and further information search. Generally, decision makers pursue a certain course of action because they believe it to be valuable. As a result, a disappointing outcome may be met with doubt — “maybe this is due to improper implementation or a random influence?” — causing decision makers to turn to others to see how their course of action has fared for them, and using this vicariously learned information to determine their next step. This is consistent with decision makers being skeptical of information that diverges from initial expectations (Levine, 1971) and also with research that performance failures represent one source of uncertainty that stimulates reliance on vicarious learning (Baum and Berta, 1999).

### 2.2 Experiential and vicarious learning in entrepreneurship

Research in entrepreneurship has highlighted that both experiential learning (Rerup, 2005; Politis, 2008; Fan et al., 2021) and vicarious learning play critical roles in shaping entrepreneurial activity (Sorenson and Audia, 2000; Nanda and Sørensen, 2010; Qin and Estrin, 2015). Experiential learning takes center stage when scholars model entrepreneurial abilities as a capability that develops with accumulating entrepreneurship experiences (Politis, 2005; Holcomb et al., 2009). Experiential learning also is central in studies examining the specific decision point to enter into or exit from self-employment (Plehn-Dujowich, 2010; Carbonara et al., 2020). Experience with their occupational choice allows entrepreneurs to learn about the payoffs associated with that choice. Armed with this knowledge, they compare these payoffs with what they aspire to earn or with what they could earn in a different choice (Gimeno et al., 1997). Subsequent choices—whether it is continuation in the current venture, exit to create a different venture, or exit to enter wage-employment—are based on this comparison, with transitions occurring when decision makers’ payoffs fall below desired thresholds (Gimeno et al., 1997; Plehn-Dujowich, 2009; Åstebro et al., 2011).

Vicarious learning similarly matters in driving entrepreneurial activity. Nikolaev and Wood (2018) argue that vicarious learning is a particularly useful strategy in the context of entrepreneurship because outcomes in this realm are uncertain, and trial-and-error processes are costly. Exiting wage-employment to give entrepreneurship a try can be a risky and involved proposition—it implies forgoing a stable income in favor of a new, risky venture that may face a failure rate of up to 90% (Patel, 2015). Looking to the experiences of others can provide at least some information for comparing alternatives without engaging in this costly trial-and-error process. In line with this, scholars find that exposure to peers that engage in entrepreneurship, or even mere observation of regionally proximate entrepreneurs, affect an observer’s entry into self-employment by shaping that observer’s confidence and career aspirations, and by providing information on road-maps, needed capabilities, and likely outcomes (Sorenson and Audia, 2000; Giannetti and Simonov, 2009; Lerner and Malmendier, 2013; Qin and Estrin, 2015; Nikolaev and Wood, 2018). Vicarious learning can also shape the reverse transitions from entrepreneurship into wage-employment. In their study on serial entrepreneurship, Nielsen and Sarasvathy (2016, p. 263) point out that entrepreneurs may not know how payoffs would change if they transitioned into wage-employment. Faced with this payoff uncertainty, it is plausible that entrepreneurs look to the experiences of their wage-employed peers and consider this vicariously learned information when making their next move. The notion that vicarious learning matters for both the transition into *and* out of self-employment is echoed in studies on how pay comparisons affect moves across a variety of occupational choices (Hartog et al., 2010; Carnahan et al., 2012).

### 2.3 Performance feedback, learning modes, and entrepreneurship

An intriguing possibility arises when we combine the evidence of entrepreneurs relying on both experiential and vicarious learning with insights from the Carnegie perspective on how performance feedback may govern the interplay between these two learning modes. Combining these lines of thought suggests that a failed venture experience—in effect, a disappointing outcome of a risky alternative—does not necessarily trigger a transition from self-employment into low-risk wage-employment. As prior research implies, if the decision maker solely relied on experiential learning, such a transition would be inevitable because adaptive learning would result in the entrepreneur choosing low-risk wage-employment in an effort to avoid future failure experiences. Yet if a disappointing payoff leads the entrepreneur to reflect and question—*I wonder if this was simply bad luck rather than an indication that entrepreneurship is an inherently poor choice? How have others fared with their ventures? And what are other options?*—exit is no longer a foregone conclusion. This is because as the entrepreneur’s doubts lead them to learn from the experiences of others, similar to how disappointing outcomes in the above railroad and baseball team examples led to vicarious learning, their next steps will be shaped by what they observe. A transition into low-risk wage-employment can still be a possible result but so is continuation in high-risk self-employment.

This similarly applies to the transition from wage-employment to self-employment. In their study on serial entrepreneurship, Spivack et al. (2014, p. 657) provide a quote from a study object who, reflecting on his repeated transition from wage- into self-employment, states that his infatuation with entrepreneurship returns as he engages with wage-employment and as “I get discontent (…) and look for something.” This comment echoes the notion that a poor payoff or dissatisfaction with a current choice motivates outward looking for figuring out next steps. Once such vicarious learning occurs, discontent with wage-employment no longer inevitably leads into self-employment. Instead, depending on what the entrepreneur observes, they may choose to continue in wage-employment for a while longer.

In what follows, we develop a formal model and employ a computational simulation to examine these learning dynamics and their outcomes more systematically.

## 3 Model

### 3.1 Background

We build on existing models of adaptive learning to formalize the choice between a risky and non-risky alternative as the result of decision makers learning experientially and vicariously (Denrell and March, 2001; Burgos, 2002; Oyarzun and Sarin, 2013). Hereafter, in accordance with us using serial entrepreneurship to illustrate our conceptualization, we use self- and wage-employment to denote risky and non-risky options, respectively. The basic model on which we build is Denrell and March (2001)’s experiential learning model. It assumes a single decision maker who chooses between a risky alternative and a non-risky one. In every period, the decision maker receives performance feedback. This feedback shapes their choice for the next period. We modify this structure to include multiple decision makers that engage in both experiential learning and vicarious learning as they select into the risky alternative (self-employment) versus the non-risky alternative (wage-employment). Our model consists of four main components: the choice between a risky- and non-risky option, payoff outcome, aspiration level, and the experiential and vicarious learning processes.

### 3.2 Self- and wage-employment as risk taking behaviors

In each period, each decision maker chooses between self- and wage-employment. For instance, a decision maker selects into self-employment with probability *p* and into wage-employment with probability 1-*p*. We use *r* to denote the case of choosing self-employment; *r* follows the Bernoulli distribution with probability *p*, i.e., Pr(*r* = 1) = *p*. To introduce vicarious learning, we assume that there are multiple decision makers and multiple periods; *p_it_* denotes decision maker *i*’s probability of choosing self-employment at time *t*. We set the initial probability of choosing self-employment at 0.5 such that at the outset of the simulation, a decision maker selects into self-employment with the same likelihood as they select into wage-employment.

### 3.3 Payoff outcome

Decision maker *i’s* payoff outcome in time *t* is denoted by *O_it_*. Our payoff captures not only monetary outcomes earned by an entrepreneur (Wright et al., 1997; Westhead and Wright, 1998) but also non-monetary utility in general. Payoff outcome is a random draw from a normal distribution. For wage-employment, the draw is from a normal distribution with a mean of *Y* and a standard deviation of zero. For self-employment, it is from a normal distribution with a mean of *X* and a standard deviation of *S*. Following Denrell and March’s (2001), and in line with Åstebro et al. (2011, p. 2015) that “the empirical literature has repeatedly revealed that self-employment earnings exhibit greater variation than wage earnings, but do not offer higher average earnings in compensation,” we set X = Y = 10, and S = 10. In later robustness checks, we vary these parameters and also experiment with drawing outcomes from a Beta distribution to model an alternative representation of the occasional extremely high or low payoffs associated with self-employment.

### 3.4 Aspiration level

The probability of decision maker *i* choosing self-employment versus wage-employment at time *t +* 1 is influenced by the decision maker comparing the payoff outcomes from their choice at time *t* with their aspiration levels. Following prior performance feedback studies and research on organizational turnover, decision makers determine their aspiration levels based on their prior payoffs (historical aspiration) or a mix of these own payoffs and those received by others (mixed aspiration) (Cyert and March, 1963; Levinthal and March, 1981; Greve, 2003; Trevor and Wazeter, 2006; Carnahan et al., 2012). Decision maker *i’s* historical aspiration at time *t*, *LH_it_,* is determined by a weighted average of their previous historical aspiration level *LH*_*i,t*-1_ and their most recent payoff outcome:


(1) $$LH_{it}=LH_{i,t-1}\left(1-b\right)+O_{i,t-1}b$$

where *b* represents a non-negative fraction denoting the weight given to the most recent outcome *O*_*i,t*-1_.

Decision maker *i’s* mixed aspiration level at time *t*, *LM_it_*, is composed of *LH_it_* and their social aspiration level at time *t, LS_it_*. *LS_it_* is computed as the mean of all decision makers’ payoffs excluding that of the focal decision maker.


(2) $$LS_{it}=\frac{1}{N-1}\underset{j\neq i}{\sum }O_{j,t-1}$$

where *j* denotes other decision makers, *O*_*j,t*-1_ the outcomes of all others at time *t−*1, and *N* the total number of decision makers. We then compute *LM_it_,* as a weighted average, with *c* denoting the weight given to the social aspiration:


(3) $$LM_{it}=\left(1-c\right)LH_{it}+cLS_{it}$$

We initially set aspiration levels at 10 and *b* = *c =* 0.5 (Denrell and March, 2001).

### 3.5 Experiential and vicarious learning

In each period, the payoffs of decision maker *i*’s choice can exceed, be sufficiently close,^1^ or fall short of their aspiration level. When payoffs exceed aspirations, the probability that decision maker *i* selects that same choice again in *t +* 1 increases (and the probability for the alternative choice decreases). For example, the choice of self-employment followed by above-aspiration payoffs at time *t* increases the probability of choosing self-employment in *t* + 1 and decreases the probability for wage-employment. This upward updating *p* is formalized as:


(4) $$p_{i,t+1}=p_{it}+a\left(1-p_{it}\right)$$

where *a* is a positive fraction that captures the speed of learning. The larger the value of *a*, the stronger the effect of a single experience on the subsequent probability of choosing self-employment. We initially set *a* = 0.4 (Denrell and March, 2001). When the choice of wage-employment is followed by an above-aspiration outcome in *t,* the probability of decision maker *i* choosing wage-employment in *t* + 1 increases, and the probability for self-employment decreases. This downward updating of *p* is formalized as:


(5) $$p_{i,t+1}=\left(1-a\right)p_{it}$$

For the case when realized payoffs in *t* are sufficiently close to the aspiration level in *t*, *p_it_* remains unchanged for *t +* 1 (Denrell and March, 2001).

**Figure 1:**
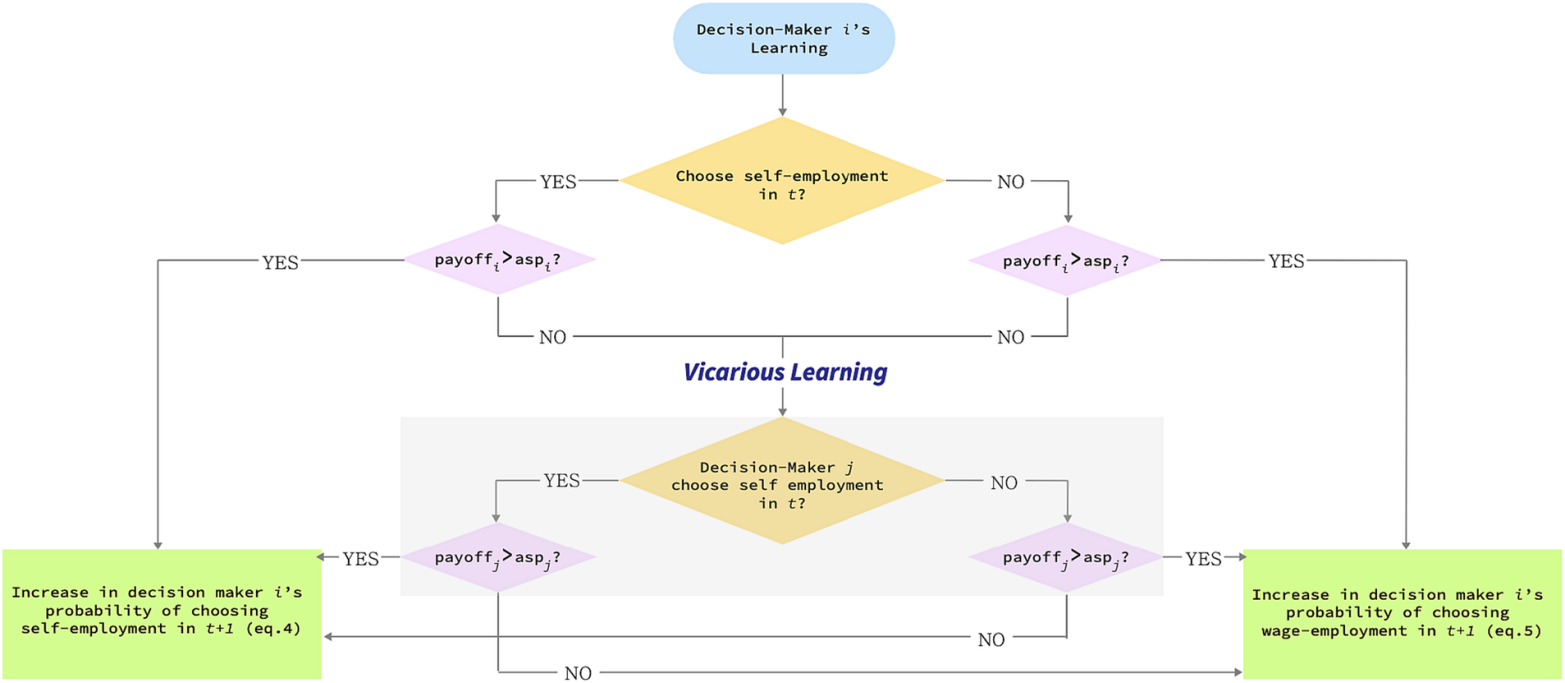
A visual representation of the learning process.

The last case is one where realized payoffs fall short of decision maker *i*’s aspirations. This triggers vicarious learning such that decision maker *i* makes their choice in *t +* 1 based on decision maker *j*’s experience in *t*. Put differently, when decision maker *i* learns vicariously, it is no longer the comparison of decision maker *i*’s payoffs with their aspirations that determines whether *p*_*i,t +* 1_ is governed by equation (4) or (5). Instead, it is decision maker *j*’s experience that determines which of the two equations governs *p*_*i,t +* 1_. In Figure 1, we provide a schematic illustration of this process.

Starting at the top of Figure 1, consider the case where decision maker *i* selects into self-employment at time *t* and receives above-aspiration payoffs. This increases the probability for decision maker *i* to re-select into self-employment in *t +* 1. Now consider the case where decision maker *i*’s venture generates below-aspiration payoffs. If decision maker *i* were to exclusively engage in experiential learning, this failure experience reduces the probability for them to re-select into self-employment and increase that of selecting into wage-employment. But in our model, below-aspiration payoffs lead decision maker *i* to look to the experience of decision maker *j*. How does this play out? If decision maker *j* also selected into self-employment and receives above-aspiration payoffs, the observation of decision maker *j*’s success experience will lead decision maker *i* to interpret their own failure experience as an unlucky fluke. Accordingly, *p*_*i,t +* 1_ is updated following equation (4), as if decision maker *i*’s own experience had been a success, and the probability of them re*-*selecting into self-employment in *t +* 1 increases. The situation differs if decision maker *j*’s payoffs from self-employment are below aspirations. This observation leads decision maker *i* to conclude that entrepreneurship is not a satisfactory option after all. Decision maker *i*’s probability of re-selecting into self-employment decreases and that of entering wage-employment increases. Instead of fully relying on *j*’s experience, as we model here, it may be possible that *i* uses *j*’s information partially or probabilistically. In a set of results not reported here, we found that our baseline results are consistent as long as the probability of using *j*’s experience is above 0.5.

To illustrate further, consider the case where decision maker *i* receives unsatisfactory payoffs in self-employment and observes decision maker *j* to engage in wage-employment where they receive below-aspiration level payoffs as well. Decision maker *j*’s unsatisfactory experience with wage-employment leads decision maker *i* to conclude that wage-employment is not a desirable choice either and, following equation (4), decision maker *i’s* probability of giving self-employment another try increases. If, instead, decision maker *j* receives satisfactory payoffs, decision maker *i* sees wage-employment as an attractive option, leading their probability of choosing wage-employment to increase as spelled out in equation (5).

Lastly, what happens if decision maker *j*’s outcome is sufficiently close to their aspiration rather than exceeding or failing it? In that case, decision maker *j* reveals ambiguous information, leaving decision maker *i* unclear whether to interpret this outcome as success or failure. Alternatively, decision maker *i* may interpret this outcome as an unconvincing success since decision maker *j*’s choice appears to be merely a satisficing alternative with payoffs that meet, but do not exceed, aspiration levels. Following prior findings that decision makers reduce reliance vicarious learning when learning targets offer ambiguous or unconvincing information (Gaba and Terlaak, 2013), we model decision maker *i* to respond to this scenario by dismissing vicariously learned information and relying, instead, on their own outcome-experience in *t* to determine the course of action for *t* + 1.

## 4 Simulation results

Results suggest that the interplay between experiential and vicarious learning centrally drives repeated transitions into and out of self- and wage-employment. For our least restrictive models—models that consider mixed aspiration levels and more than two decision makers—we find that out of 500 instances in which a transition back into self-employment could occur, the itch strikes in 138 of those, a rate of 27.6%. In our baseline models—models with historical aspiration levels in a two-actor setting—the entrepreneurial itch strikes less frequently. Nonetheless, we begin by presenting these baseline models because they most readily reveal the exact processes that underlie the recurrence of the entrepreneurial itch.

### 4.1 Intermittent risk taking

**Figure 2:**
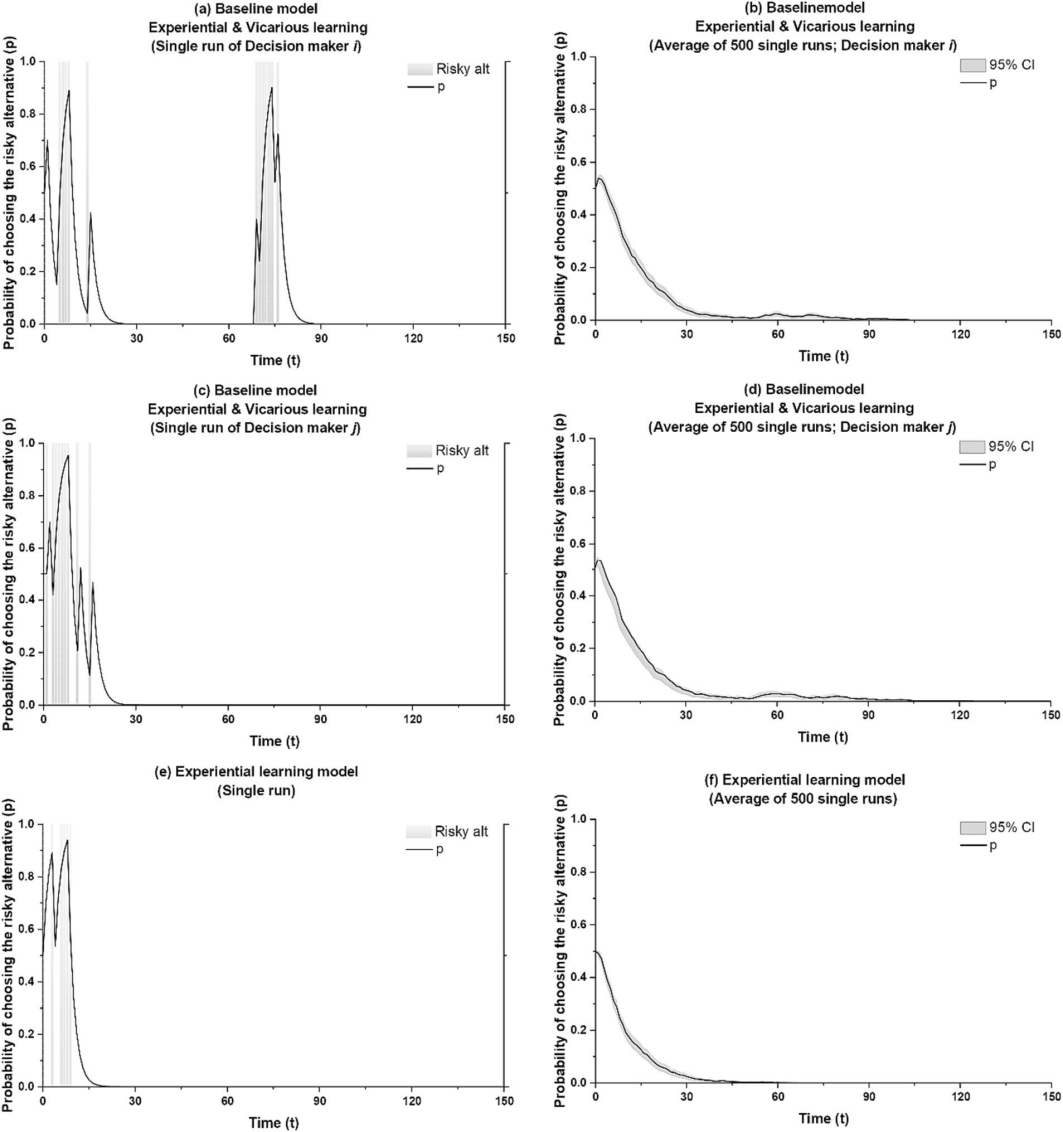
Emergence of recurring itches with vicarious learning. The shaded areas in **(a**,**c)** and **(e)** indicate actual risk-taking.

In our baseline models, the interplay between experiential and vicarious learning leads some entrepreneurs, though not all, to return into risk taking subsequent to engaging in the non-risky option. Figure 2A shows a single simulation run for this pattern. The decision maker for whom the itch strikes is denoted as decision maker *i* and the other as decision maker *j*. Shaded areas in Figure 2 indicate when a decision maker engages in self-employment, i.e., where *r* = 1. Starting with a 50% probability of selecting into self-employment at the outset of the simulation, Figure 2A shows that decision maker *i* initially gives entrepreneurship a try, only to settle, seemingly for good, into wage-employment subsequent to period 25. A selection into the low-risk option aligns with adaptive learning where experiential learning from an occasional extreme failure experience with the risky option guides the decision maker to settle with the lower-risk option (Denrell and March, 2001; Burgos, 2002; Oyarzun and Sarin, 2013). Yet it plays out differently in our model: in Figure 2A, *p* sharply increases to nearly one around period 69, indicating that the decision maker is likely to return into self-employment for a while—the entrepreneurial itch has struck. The pattern in Figure 2A—i.e., the return into self-employment after a period in wage-employment—is representative of 12% of all baseline-model runs that start out with an initial selection into entrepreneurship.

In our baseline models, intermittent risk taking occurs one time only. Put differently, if risk taking recurs once, there are no other sharp increases in *p* for that decision maker within the next 2,000 periods. (We end the horizontal axis in Figure 2 at *t* = 150 only to facilitate illustration). What is more, for any given run in which risk taking recurs, it only recurs for one of the two decision makers and never for both. Figure 2C depicts decision maker *j*’s pattern for the simulation run shown for decision maker *i* in Figure 2A. After the first wave of risk taking, decision maker *j* settles into wage-employment and remains there for the next 2,000 periods. This pattern—risk taking recurring for decision maker *i or j*, but not both—is representative of 100% of all baseline-model cases.

Figures 2B,D show averaged result of 500 independent simulation runs for decision maker *i* and *j*, respectively. Intermittent risk taking is less pronounced in this averaged presentation but notable as a small uptick in *p* after *t* = 50. Of course, these averaged results understate the importance of recurring risk taking. Consider that only 50% of decision makers initially engage in the risky alternative, thereby setting the necessary stage for the itch to strike again at a later point. Also consider that not every initial entrepreneur re-engages in self-employment after settling into wage-employment, and that for those for whom the itch strikes, it strikes at different times. Ultimately, out of 500 independent simulation runs, risk taking recurs in 60 and 62 runs for decision maker *i* and *j*, respectively.

To clarify the role of the interplay of vicarious and experiential learning in the recurrence of risk taking, we compare our results with those from a model where decision makers solely learn experientially (Denrell and March, 2001). In such a model, a failure experience in self-employment reduces the probability of re-selecting into self-employment according to equation (5), and unsatisfactory payoffs in wage-employment reduce the probability for re-selecting into wage-employment according to equation (4).

Figure 2E shows a single simulation run for this experiential learning-only model. Mirroring the run shown in Figure 2A, a decision maker initially selects into self-employment. However, self-employment’s confluence of the occasional high payoff that drives up aspirations and an eventual failure experience that does not meet these aspirations soon guides the decision maker into wage-employment, consistent with Denrell and March (2001). Unlike in our baseline model, the decision maker, once settled in wage-employment, does not re-select into self-employment at a later point. The pattern shown in Figure 2E is representative of 100% of runs. Averaged results for the experiential learning-only model, shown in Figure 2F, reflect this, with no noticeable uptick in *p* in later periods.

We use *t*-tests to examine whether the difference in the occurrence of intermittent risk taking between our model and the experiential learning-only model is statistically significant. We consider both the likelihood of recurring risk taking as well as each incident’s duration. We capture re-engagement in self-employment to take place when *p* bounces back up to near one subsequent to having reached a point below 0.001. Duration is captured by the number of periods between the point of initial departure of *p* from below 0.001 and the time of return of *p* to below 0.001. Results, presented in Table 1 [Line (B)], show that our model of experiential and vicarious learning produces a statistically significant higher number of the entrepreneurial itch recurring than the experiential learning-only model (mean = 0.122 [61 cases], s.d. = 0.330 versus mean = 0.000 [0 cases], s.d. = 0.000, respectively).

**Table 1:** Differences in recurring itches between models (*t*-test).

	Likelihood of recurrence			Likelihood of recurrence (Both decision makers)			Duration		
	Mean	SD	*t*-statistics	Mean	SD	*t*-statistics	Mean	SD	*t*-statistics
(A) Experiential learning only	0.000	0.000							
(B) Baseline (Historical aspiration)	0.122	0.330	11.673*** (B) – (A)	0.000	0.000		20.033	8.632	
(C) Mixed aspiration	0.115	0.340	−0.467 (C) – (B)	0.648	0.482	30.294*** (C) – (B)	28.361	15.049	5.204*** (C) – (B)
(D) Four-actor historical aspiration	0.128	0.338	0.461 (D) – (B)				20.822	9.454	0.776 (D) – (B)
(E) Four-actor mixed aspiration	0.276	0.533	8.707*** (E) – (C)				49.065	37.979	5.564*** (E) – (C)

Likelihood of recurrence = number of recurring incidences/total number of independence simulation run. *p < 0.1, **p < 0.05, ***p < 0.01.

Taken together, these results indicate that the interplay between experiential and vicarious learning is key for repeated transitions into and out of self-employment. Without vicarious learning, risk taking does not recur. Yet results also suggest that while this learning-mode interplay is central for the entrepreneurial itch to strike, it does not lead all entrepreneurs to reselect into self-employment. What, then, are the exact circumstances in which this learning interplay triggers the transition back into self-employment?

### 4.2 Dynamics of intermittent risk taking

Intermittent risk taking comes about as two circumstances converge: The first creates a situation where the focal decision maker, upon experiencing a failure in self-employment, has a learning target that contentedly engages in wage-employment. Through learning vicariously, this leads the focal decision maker to engage in wage-employment as well. The second circumstance makes for a situation where the learning target eventually becomes unconvincing, causing the focal decision maker to revert back to learning experientially and giving self-employment another try. We shed light on each circumstance below.

The first circumstance is a result of one decision maker, here decision maker *i,* experiencing an early success in self-employment whereas decision maker *j* experiences an early failure. For decision maker *i,* this success increases their aspirations. Of course, heightened aspirations increase the likelihood for a subsequent unsatisfactory outcome. This outcome leads decision maker *i* to question whether entrepreneurship is the right choice after all, and to look to decision maker *j* to inform their next step. As for decision maker *j*, their early failure in entrepreneurship lowered their aspirations. This increased the chances of satisfactory payoffs even if engaging in wage-employment, and their selection into wage-employment solidifies.^1^ As decision maker *i* observes decision maker’s *j* satisfaction in wage-employment, they transition into wage-employment as well.

What happens next? Absent the second circumstance, both decision makers tend to stay in wage-employment from here on out. But consider what happens if decision maker *j* becomes an unconvincing learning target. After following decision maker *j* into wage-employment, decision maker *i* continues to learn vicariously. This is because decision maker *i*’s initial entrepreneurial success increased their aspirations. These lingering aspirations outpace the payoffs from wage-employment, leading decision maker *i* to be continually uncertain about the right course of action and to learn from decision maker’s *j* experiences. This keeps decision maker *i* in wage-employment as long as decision maker *j*’s experiences make wage-employment appear attractive. But this changes when decision maker *j*’s signal becomes ambiguous. As decision maker *i* observes that decision maker *j*’s payoffs are merely near aspirations, decision maker *j* becomes an unconvincing learning target: perhaps decision maker *j* has been pursuing wage-employment simply because this is what they have been doing in the past rather than because it is a truly satisfying option. With no other suitable target to learn from, decision maker *i* reverts back to learning experientially and relying on their own aspiration-outcome comparison to guide next steps. Since these aspirations are still higher than what wage-employment can deliver, decision maker *i* tries their hand at self-employment again—the entrepreneurial itch that drives the decision maker into intermittent risk taking is back.

**Figure 3:**
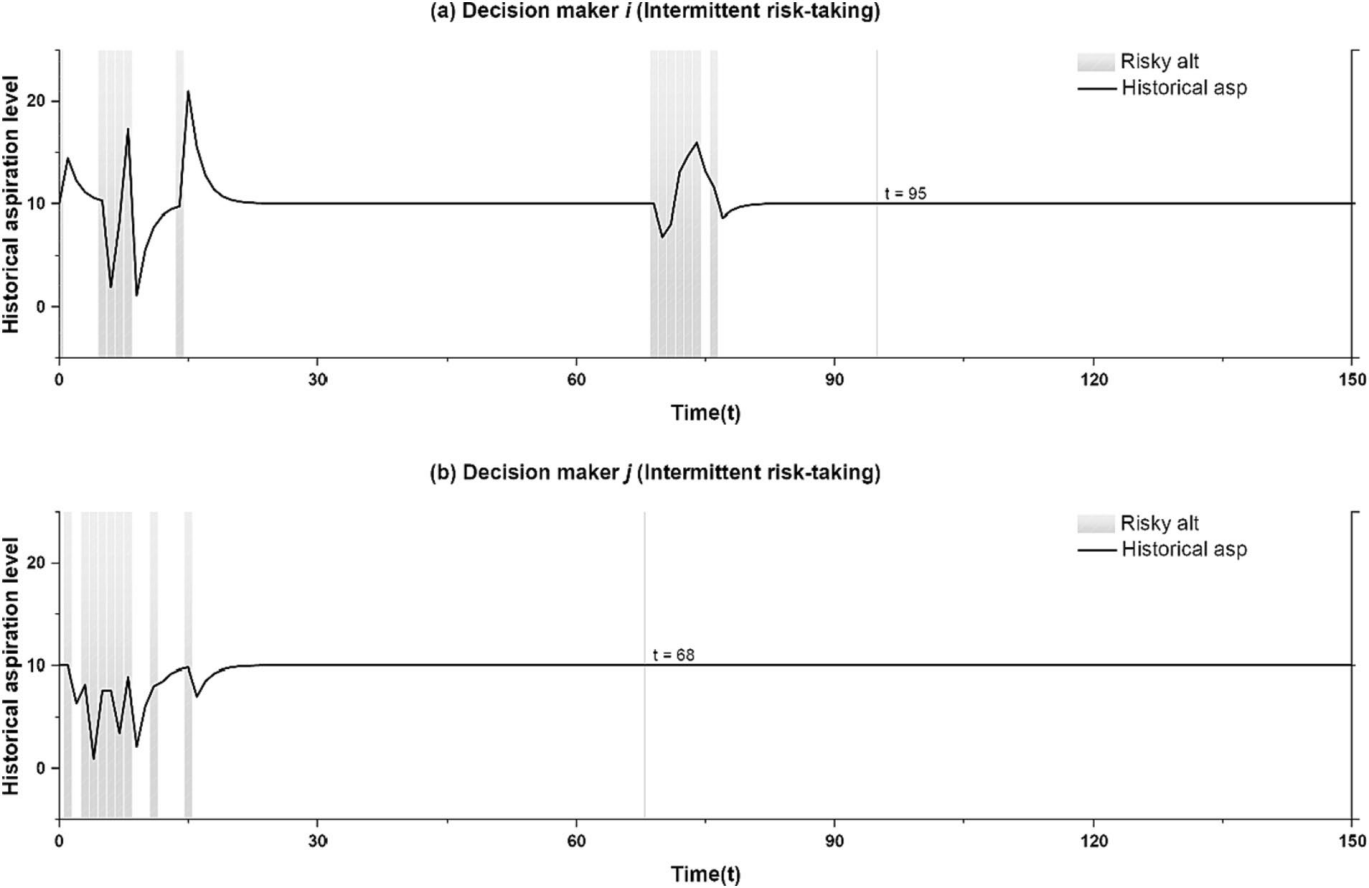
The role of aspiration in generating recurring itches. The shaded areas in **(a,b)** indicate actual risk-taking.

For the above process to unfold, the timing at which wage-employment becomes but a satisficing option is important: in the above case, it needs to happen sooner for decision maker *j* than for decision maker *i.* This occurs quite frequently; all it requires is that decision maker *j*’s failure experience with entrepreneurship is less severe and earlier than decision maker *i*’s initial success experience. To elaborate more, we use Figure 3 which shows variations in aspiration levels of the two decision makers across time. Figures 3a,b are tied to the run shown in Figures 2a,b, with Figure 3a showing how decision maker *i’s* aspirations move along as the run from Figure 2a unfolds, and Figure 3b showing corresponding changes in decision maker *j*’s aspirations.

In Figures 3a,b, both decision makers’ aspiration levels converge toward 10, but total convergence happens sooner for decision maker *j* (at *t* = 68) than decision maker *i* (at *t* = 95). To understand why this is, note that in the run shown in Figures 3a,b, decision maker *i* had a success event that occurred a bit later and was more extreme than the initial failure event experienced by decision maker *j*. With aspirations adapting at the same rate for both decision makers, this results in a situation where total convergence between aspirations and expected outcomes occurs sooner for decision maker *j* than for decision maker *i*. Specifically, in period 68, decision maker *i* observes that for decision maker *j,* wage-employment has become a satisficing option with payoffs merely meeting aspirations. At that time, decision maker *i*’s aspirations still outpace the payoffs from wage-employment. It is this precise combination of decision maker *j* revealing ambiguous information about the attractiveness of wage-employment and decision maker *i* finding that its payoffs still fail to meet their own aspirations that prompt them to give entrepreneurship another try.

### 4.3 Extended model with mixed aspirations

Results so far suggest that intermittent risk taking occurs when decision makers engage in an interplay between experiential and vicarious learning, and, further, when initial failure and success experiences align such that the appeal of wage-employment, as signaled by decision maker *j*, becomes unconvincing prior to decision maker *i* fully resigning themselves to wage-employment as a satisficing choice. We next explore how the frequency of this confluence of events changes when decision makers form their aspirations not only based on their own experiences but also the experiences of others, as spelled out in equation (3).

When we account for mixed aspirations, intermittent risk taking can occur for both decision maker *i and j* in a given run, rather than just one of them. This comes about because decision maker *j,* subsequent to their initial entrepreneurial failure and selection into wage-employment, now incorporates decision maker *i*’s early entrepreneurial success when forming their own aspirations. Since this will upward-adjust decision maker *j’s* aspirations, these aspirations begin to outpace wage-employment’s payoffs. This prompts decision maker *j* to learn vicariously from decision maker *i*, who, at that point, is happily engaged in self-employment. As a result, decision maker *j* re-selects into entrepreneurship as well.

**Figure 4:**
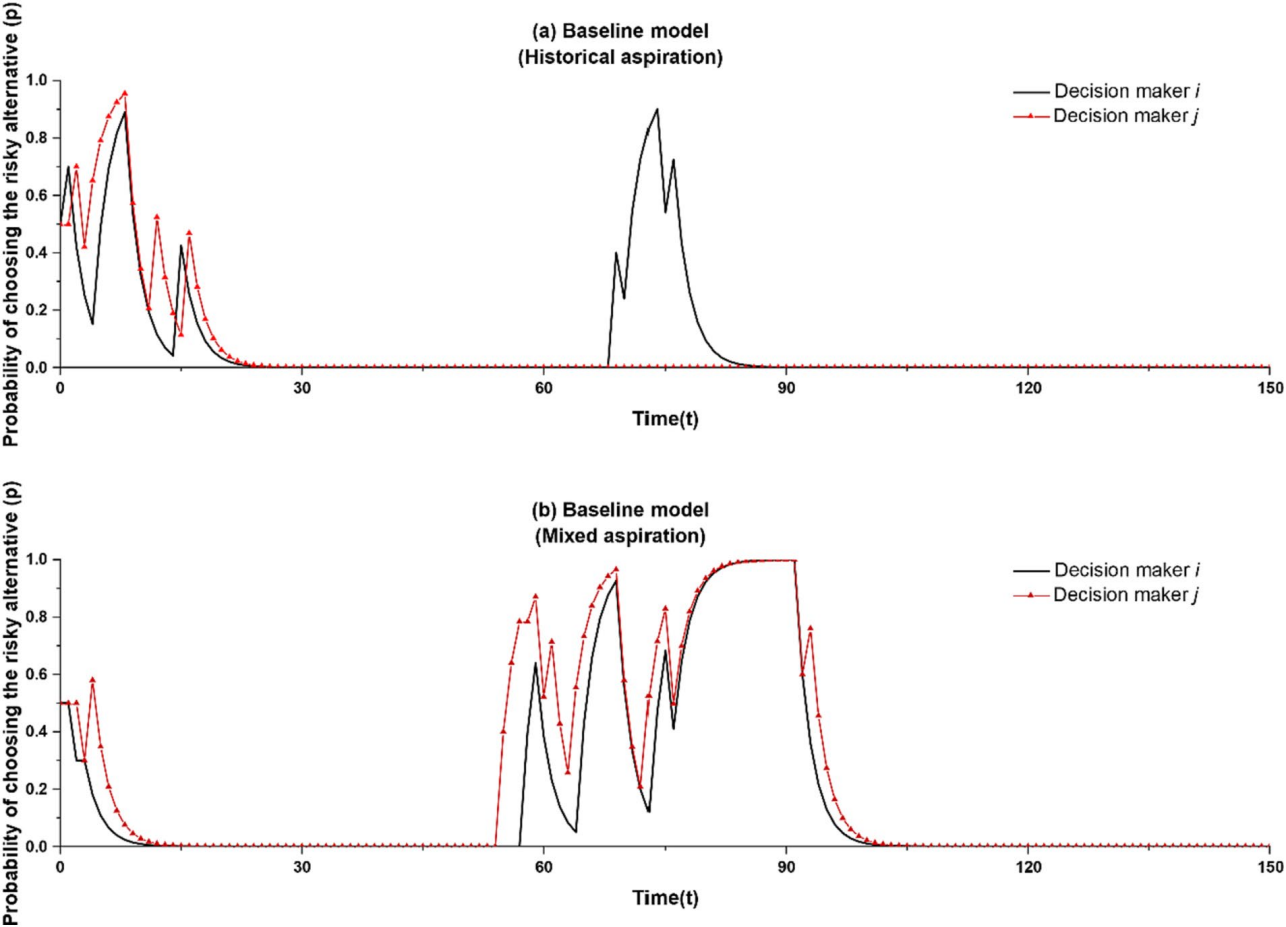
**(a)** and **(b)** illustrate intermittent risk-taking with historical and mixed aspiration, respectively.

Figure 4 illustrates these results. Similar to Figures 2a–c, it shows individual simulation runs. Yet different to Figures 2a–c, it shows the probability of the two decision makers selecting into self-employment across time. Figure 4a shows a run from our baseline model with historical aspiration levels, with risk taking recurring to only one of the decision makers (in this case, decision maker *i*). Figure 4b shows a run from a model with mixed aspiration levels. Here, the pattern of the itch striking both decision makers in the same run is representative of 44% of all runs in which the itch strikes.

We conduct *t*-tests to analyze statistically how the recurrence of risk taking differs between the baseline model and mixed aspirations model. Results, presented in Table 1 [Line (C)], show that the total number of times with which risk taking recurs is not significantly different between the two models. However, the likelihood of *both* decision makers re-selecting into self-employment is significantly higher in the mixed aspirations model than the baseline model (mean = 0.648 [35 cases], s.d. = 0.482 versus mean = 0.000 [0 cases], s.d. = 0.000, respectively). What is more, in the mixed-aspirations model, decision makers re-engage in self-employment for significantly longer than in the baseline case (mean = 28.361 per risk taking, s.d. = 15.049 versus mean = 20.033 per risk taking, s.d. = 8.632, respectively).

### 4.4 Extended model with multiple decision makers

**Figure 5:**
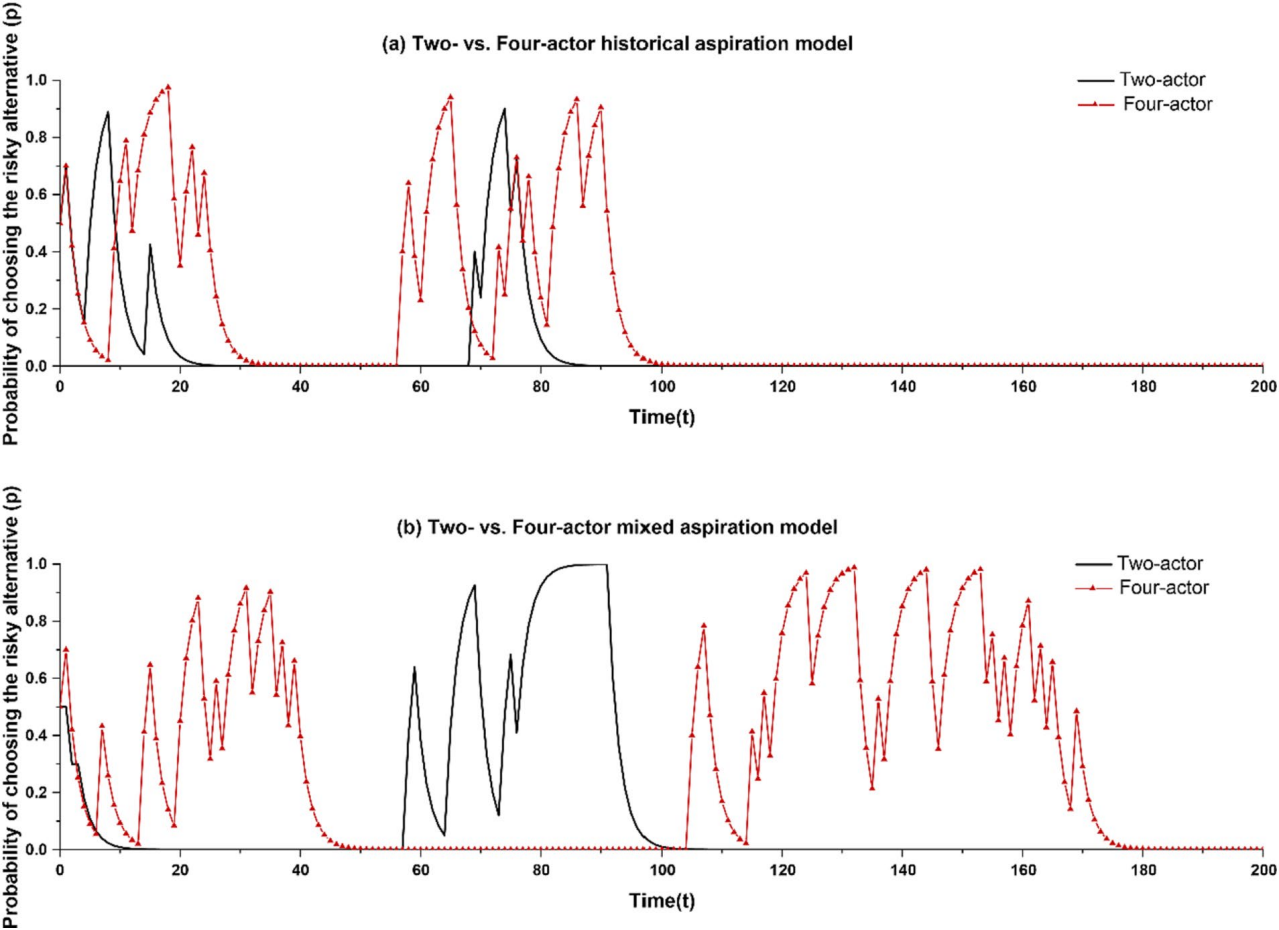
Comparison between two-actor and four-actor models. The two-actor models in **(a,b)** correspond to the baseline model and the mixed aspiration model, respectively.

As a final model iteration, we increase the number of decision makers beyond two. We assume that when decision makers learn vicariously subsequent to an unsatisfactory outcome, they select their learning target through a tournament selection mechanism whereby a subset of *m* decision makers is randomly chosen from the population of *N* (Posen et al., 2013). The learner compares the payoffs of these randomly chosen decision makers and selects the decision maker with the highest payoff as learning target. After this selection process, the learner follows the vicarious learning rule specified earlier. To capture the net effect of increasing the number of decision makers, we initially set *m* = 1 and *N* = 4. With *m* = 1, the selection process is random, allowing us to attribute differences in this case to the increase in population from two to four. We vary the value of *N* in later robustness checks. In Figure 5, we report typical runs from the four decision maker-model as compared to the two decision maker-model with historical aspirations (Figure 5a) and mixed aspirations (Figure 5b).

We find that in the four decision maker-model with mixed aspiration levels, intermittent risk taking becomes very pronounced, both in terms of the number of times decision makers return into self-employment as well as how long they stay with this selection. Of all cases in which intermittent risk taking occurred at least once (in our analysis, this is in 138 out of 500 instances), risk taking recurs at least once in 73.96% of these cases, and more than once in 26.04% of cases.

How does this difference come about? Recall that in the baseline model, the entrepreneurial itch struck and risk taking recurred to decision maker *i* as decision maker *j*’s signal about the attractiveness of wage-employment became ambiguous. This prompted decision maker *i* to reckon with their own aspirations still outpacing the payoffs of wage-employment, leading them to give self-employment another try. Yet in the baseline model, the entrepreneurial itch was a one-time occurrence: because decision maker *j* had become an unviable learning target, decision maker *i* learned only experientially from that point onwards. This exclusive reliance on experiential learning eventually led the decision maker to select into wage-employment for good. This is where the multiple decision maker-model differs: decision maker *i*, subsequent to their second bout with entrepreneurship, can choose alternative learning targets, thereby restarting the process that led to the initial recurrence of the itch.

We conduct *t*-tests to examine statistical differences in the results of the two models. Table 1 [Line (E)] shows that the model with four actors and mixed aspirations produces significantly more returns into self-employment (mean = 0.115 [57.5 cases], s.d. = 0.340 for the two-actor model versus mean = 0.276 [138 cases], s.d. = 0.533 for the four-actor model). The self-employment bouts are also of greater duration (mean = 28.361 per risk taking, s.d. = 15.049 for the two-actor model versus mean = 49.065 per risk taking, s.d. = 37.979 for the four-actor model). However, when we consider historical aspirations in the four-actor model [Line (D)], the four- versus two-actor models generate a similar number of recurrences (with mean = 0.122 [61 cases], s.d. = 0.330 for the two-actor model versus mean = 0.128 [64 cases], s.d. = 0.338 for the four-actor model). There also is no significant difference in the duration of the risk taking (with mean = 20.033, s.d. = 8.632 for the two-actor model versus mean = 20.822, s.d. = 9.454 for the multi-actor model).

### 4.5 Sensitivity analysis

We examine the sensitivity of our results to changes in parameter values and the rules governing vicarious learning. As for altering parameters, we vary the speed of learning (parameter *a*), the rate with which aspirations adapt to outcomes (parameter *b*), and the weight given to others’ aspirations when decision makers use mixed aspirations (parameter *c*). As one might expect, when learning is very slow (*a* = 0.1), *p* adapts very slowly, and the switch between self-employment and wage-employment becomes rare. In effect, very slow learning results in decision makers sticking with their initial choice, thereby limiting the opportunity for the itch to strike. Yet beyond this, results are robust across a considerable range of values for *b* and *c,* with any differences in timing or degree of the entrepreneurial itch being too minor to qualitatively affect results.

We also vary *m*, the size of the sub-sample for tournament selection. We find that as the sub-sample for the tournament selection increases, the general tendency to select into self-employment increases, thereby limiting the recurrences of risk taking. In other words, intermittent risk taking occurs less frequently because decision makers display a more general and prevailing tendency to engage in self-employment—the itch cannot recur as much because it does not go away as often in the first place. A primary driver for this is the tournament selection mechanism whereby decision makers vicariously learn from the best performing learning target in the chosen sub-sample. In our model, these high performers are successful entrepreneurs. As their success with self-employment promotes entrepreneurship to those that learn vicariously, a population-level propensity to select into self-employment emerges. As this selection prevails, a prerequisite for intermittent risk taking—near-convergence of *p* to 0—occurs less often. We also vary *N*, the number of decision-makers in the population, to 5, 7, and 9. In all cases, we found significantly higher likelihoods of recurring risk taking with longer duration than in models that consider only experiential learning.

A final modification in parameter values relates to assigning each decision maker a randomly drawn value for *p*, with all values coming from within the range of 0.1 to 0.9 (i.e., *p_t = 0_ =* 0.1 ~ 0.9). No major difference in results emerges as a result of this modification.

We also experiment with payoff distributions other than the normal distribution. There is a high rate of failed startups and extremely few ventures that become “unicorns,” implying that payoff distributions may be skewed with a significant probability mass on the left side. To account for this, we adopt a beta distribution that follows this tendency. We keep the mean and the standard deviation at 10. Under this alternative characterization of self-employment payoffs, in both historical- and mixed-aspiration cases, the entrepreneurial itch recurs similarly to how it does in the results from models using the normal distribution.

Lastly, we test the robustness of our results to changes in the vicarious learning process. In our model, decision makers cease to learn vicariously when the learning target’s payoffs equal their aspirations. When observed payoffs neither exceed nor fall below aspiration levels, revealed information becomes ambiguous, making it difficult for the observer to conclude whether the focal alternative is attractive. Research suggests that when learning targets reveal such ambiguous information, decision makers reduce their reliance on vicarious learning (Gaba and Terlaak, 2013). It also is plausible that the observer stops learning vicariously because while payoffs may still be satisficing, they insufficiently endorse the focal alternative and hence are unconvincing. This pathway is different from the first: it is not about ambiguity but about the observer deciding that an alternative that generates but satisficing payoffs is not worth pursuing. Testing the sensitivity of our results to changes in this learning rule shows that our results require that decision makers assess an alternative as attractive only when its payoffs exceed aspirations and not when its payoffs meet aspirations.

We also explore if results are sensitive to the assumption that when learning vicariously, decision makers rely entirely on their learning target’s experience for informing their own course of action. As an alternative, we model the focal decision maker’s reliance on the learning target’s information to be governed by a randomly assigned probability between 0 and 1. Results for this case are qualitatively similar to the ones we report here.

## 5 Discussion

The antecedents and consequences of risk taking in organizational life is a central area of inquiry in the Carnegie perspective (Cyert and March, 1963). In this study, we draw on organizational learning literature and use the context of entrepreneurship to develop a computational model that illuminates the mechanisms that can underlie time-varying risk taking tendency. In particular, we delineate conditions under which abrupt risk taking punctuates periods of risk-avoiding behaviors, a pattern that we call “intermittent risk taking.” We use serial entrepreneurs whose bouts with risk taking are often depicted as driven by an “entrepreneurial itch” to illustrate our model. Reflecting on their repeated move between self- and wage-employment, one entrepreneur in Spivack et al.’s (2014, p. 657) study explains that “when I close down a venture for whatever reason, if it fails or just misses the mark and I go back to the corporate world, […] it does not last long. After 2 years, I’m just itching to do something else and it shows on my resume.” We have shed light on learning mechanisms that can explain this pattern of entrepreneurs repeatedly moving back and forth between risky and non-risky alternatives, and, further, have examined the exact circumstances under which the itch for risk taking recurs.

Using a computational model that conceptualizes decision makers to chart their course by learning from their own experiences as well as those of others, and using our serial entrepreneurship illustration, the following storyline emerges. The repeated switch between self-employment and wage-employment, i.e., intermittent risk taking, comes about as an entrepreneur, primed by early success in venture creation, develops too high expectations about the payoffs that entrepreneurship can consistently deliver. Once disillusioned, this entrepreneur looks to their peers who, after initial failures in venture creation, found success in wage-employment. Encouraged by this observation, the entrepreneur engages in wage-employment as well. Yet over time, these peers become less convincing as they begin to pursue wage-employment as a satisficing option, rather than one that is truly fulfilling. Absent any observations that make wage-employment unambiguously attractive, and with their early venture success having left a lingering expectation that payoffs should be higher than what wage-employment can deliver, the entrepreneur gives venture creation another try. The entrepreneurial itch is back.

This mechanism for intermittent risk taking has a number of implications for research on varying risk taking preferences, serial entrepreneurship, and theories of organizational learning generally.

### 5.1 Risk taking and organizational learning

Our model of adaptive learning driving the selection into high-risk self-employment versus low-risk wage-employment builds on prior conceptualization of organizational learning and risk taking in the Carnegie perspective (Cyert and March, 1963; Denrell and March, 2001; Denrell, 2008; Oyarzun and Sarin, 2013). Prior work in this perspective elucidates that experiential learning fosters a bias against risk. This bias comes about because risky alternatives have extreme outcomes, both good and bad, with the eventual bad outcome prompting decision makers to shy away from subsequent risks (March, 1996; Denrell and March, 2001). Vicarious learning is associated with an opposite, risk-seeking bias. When learning vicariously, decision makers often learn from samples that are biased toward observations that are enjoying success with risky alternatives, thereby prompting observers to become risk seeking as well (Denrell, 2003). Vicarious learning can also attenuate, though typically not overturn, the bias against risk that emerges from experiential learning since it can enable decision makers to access information about foregone outcomes from the risky alternative (Denrell and Le Mens, 2007; Smith and Collins, 2009).

When contemplating how risk taking may then unfold when the two learning modes interplay, a reasonable ex-ante expectation would be that experiential learning will drive decision makers into the low-risk alternative, only for vicarious learning to subsequently pull them back into the high-risk choice. But this is not what our results suggest, at least not in the context of serial entrepreneurship. To be sure, results do show that vicarious learning is instrumental for the repeated selection into the high-risk choice, i.e., venture creation—there was no evidence of such intermittent risk taking when decision makers engaged in experiential learning only. Yet rather than prompting a return into the risky choice by having entrepreneurs observe a lopsided sample of successful risk takers, it prompted this return as the focal entrepreneur observed that for others, the low-risk option (wage-employment) was merely a satisficing option, rather than a fulfilling one. This suggests some interesting avenues for future research: it hints that once we allow for different learning modes to interplay, each individual mode may drive risk taking in ways that are more nuanced than what studies that examine each learning mode in isolation have shown.

### 5.2 Interplay of experiential and vicarious learning

Prior entrepreneurship research has heavily drawn on theories of organizational learning to explain both occurrence and success of entrepreneurial activity (e.g., Corbett, 2005; Lumpkin and Lichtenstein, 2005; Rerup, 2005). Yet for the most part, this research has emphasized one learning mode over the other (see Lévesque et al., 2009 for an exception) and has left underexplored how these learning modes can interplay to affect entrepreneurship. We have drawn on organizational learning studies from the Carnegie perspective (and from outside the entrepreneurship realm) to address this gap. Building on conceptualizations by Baum and Dahlin (2007) and Schwab (2007), we model decision makers to engage in experiential learning when performance outcomes exceed aspirations and in vicarious learning when they fall below aspirations. Two implications emerge: First, accounting for decision makers to engage in a combination of learning modes can explicate patterns of entrepreneurship and risk taking that are difficult to explain by either learning mode alone. With experiential learning leading to risk aversion (and hence the eventual abandonment of high-risk choices) and vicarious learning encouraging risk seeking, it is unclear that either learning mode can fully explicate phenomena that involve repeated switches between high- and low-risk options. While we have focused on how a learning mode combination can drive the repeated switch between self- and wage-employment, future research could explore how such a combination can account for other entrepreneurial behaviors with varying risk taking tendencies.

A second implication relates to the importance of the mechanism that governs the interplay between learning modes. In our study, this mechanism centrally influences results, thereby highlighting the importance of analyzing what the effects of other governing rules might be. The mechanism in our model rests on the notion that disappointing experiences seed doubt about the appropriate path to pursue, thus prompting a reliance on learning from others (Baum and Dahlin, 2007; Schwab, 2007; Aranda et al., 2017; Clough and Piezunka, 2020). Yet there may be alternative ways in which the learning modes interplay. We hope that future studies will explore these ways and their effects on behaviors.

### 5.3 Implications for entrepreneurship

Entrepreneurship is often not a lifelong commitment. Instead, it frequently involves transitions between self-employment and wage-employment (Koch et al., 2021; Feng et al., 2022). Our research both complements and extends existing insights into shifting patterns in entrepreneurial careers by suggesting a mechanism that elucidates when and why these transitions occur. Our computational results are similar to empirical patterns that capture “mixed self-employment career patterns” (Koch et al., 2021, p. 8). In our simulated scenario with multiple decision makers, we found 28% of decision makers to transition between self- and wage-employment; in their empirical analysis, Koch et al. (2021) show such a pattern for 33% of their sample. This alignment between empirical and computational findings lends confidence in applying our mechanism to understanding the recurrent risk taking inherent in serial entrepreneurship.

Our findings hint that the pursuit of entrepreneurship can come about simply because it is the next-best option. In other words, decision makers may (re)engage in self-employment not because they discover an opportunity or an opportunity presents itself, as stressed by studies exploring opportunity-led entrepreneurship (Baron and Ensley, 2006; Aparicio et al., 2016), and also not because they perceive self-employment as the occupation that offers higher expected payoffs, as other studies emphasize (e.g., Holmes and Schmitz, 1990; De Wit, 1993). Instead, (re)engagement in entrepreneurship comes about as decision makers fail to discover wage-employment to be a satisfactory alternative.^1^ This speaks to the literature on necessity entrepreneurship, and, specifically, to recent conceptualizations of necessity entrepreneurship as lying along a continuum of needs. On one end of this continuum, decision makers engage in entrepreneurship because they lack other options to meet basic needs whereas on the other end, they pursue entrepreneurship as a means of fulfilling higher-level needs (Coffman and Sunny, 2021; Dencker et al., 2021). Our study addresses the middle ground between these two ends: we propose that decision makers engage in self-employment as a next-best option simply because it is expected to better meet their aspirations than wage-employment. As future studies further investigate the drivers of this type of entrepreneurship, it will be interesting to examine how a learning-based explanation like ours combines with other drivers to provide a more complete understanding of the settings and mechanisms underlying these nuances in entrepreneurship.

Implications also emerge from our analysis of the timing of the entrepreneurial itch. Assuming that venture creation opportunities exist at any point in time, what are the mechanisms that determine when an entrepreneur jumps on them? In extant necessity-based entrepreneurship research, this timing tends to be determined by broader economic conditions and individual attributes that foreclose other employment options (Poschke, 2013; Brush et al., 2017; Fairlie and Fossen, 2018). In contrast, in our framework, the timing of the entrepreneurial itch is determined by the point at which vicarious learning leads the decision maker to become unconvinced about the attractiveness of wage-employment. The notion that the timing is shaped by a threshold consideration (i.e., by some comparison between payoffs and expectations) aligns, in principle, with Gimeno et al.’s (1997) study on entrepreneurial exit. In that study, the switch from venture creation into something else also is based on a threshold consideration—it occurs when the payoffs of entrepreneurship fall below a certain level. For Gimeno et al. (1997), the location of this threshold is determined by the payoffs that the decision maker could receive from foregone alternatives, which, in turn, depend on their human capital attributes. This is where our study differs. In our framework, the threshold is determined by a vicarious learning process, with vicarious learning prompting a switch back into venture creation when it leads the focal entrepreneur to realize that the payoffs from wage-employment are only just good enough to keep others in this alternative, but not good enough for their own aspirations. This hints that factors other than human capital and economic conditions may shape the disappointment threshold that triggers a switch into (or out of) self-employment. We draw attention to the role that social processes like vicarious learning may play; it is worthwhile exploring how other socially constructed thresholds may drive patterns of (serial) entrepreneurship.

Lastly, while our study focuses on transitions between self-employment and wage-employment, it is important to acknowledge that entrepreneurs might also pursue various other states, such as vocational training and unemployment, as highlighted by Koch et al. (2021). Each of these categories may be associated with unique risk taking propensities, offering a promising avenue for additional investigation. Specifically, future research can use a risk taking framework to analyze further how decision makers transition between these different employment states, thereby providing a more comprehensive understanding of the mechanisms underlying the multifaceted aspects of entrepreneurial career choices.

## 6 Conclusion

Departing from traditional utility-curve-based explanations for risk taking, the Carnegie perspective proposed a set of behavioral approaches for understanding risk taking (Cyert and March, 1963; March and Shapira, 1992). To this day, these behavioral approaches continue to shape an important body of research in the field. In this study, we have continued in this line of inquiry to develop a learning based model that illuminates the processes that can underlie recurrent risk taking. Illustrating our model with serial entrepreneurs repeatedly transitioning between high-risk self-employment and low-risk wage-employment, we show that an interplay of experiential and vicarious learning can drive the type of intermittent risk taking inherent in repeated venture creation. Our study points to a promising line of inquiry examining mechanisms for varying risk taking tendencies and serial entrepreneurship from an organizational learning perspective.

## Data availability statement

The raw data supporting the conclusions of this article will be made available by the authors, without undue reservation.

## Author contributions

All authors contributed to the article and approved the submitted version.

## Footnotes

^1^Some entrepreneurs called "portfolio entrepreneurs" are involved in multiple startups at the same time.

^1^Throughout the manuscript, we use "they" and "their" as a gender-neutral pronoun for decision makers and entrepreneurs.

^1^We say payoff and aspiration level are sufficiently close when the absolute difference is less than 1 x 10^-4^.

^1^Our models are probabilistic. Thus, while decision maker *j*, in response to their early entrepreneurial failure, learns vicariously from decision maker’s *i* entrepreneurial success, and while this increases *p* *_jt+1_*, there still is a chance that decision maker *j* engages in wage-employment in *t*+1. When this happens, their lowered aspirations make the payoffs of wage-employment satisfactory, causing decision maker *j* to settle into this selection.
